# The Effect of Initial Microstructure and Hardenability on Diode Laser Surface Hardening of Medium-Carbon Steels

**DOI:** 10.3390/ma19050981

**Published:** 2026-03-03

**Authors:** Lyubomir Lazov, Edmunds Teirumnieks, Gatis Muiznieks, Armands Leitans, Jiří Čapek, Karel Trojan, Prodan Prodanov, Emil Yankov, Normunds Teirumnieks, Ritvars Rēvalds, Imants Adijāns

**Affiliations:** 1 Rezekne Academy of Riga Technical University, Atbrivosanas Aleja 115, LV-4601 Rezekne, Latvia; edmunds.teirumnieks@rtu.lv (E.T.); emil.yankov@rtu.lv (E.Y.); ritvars.revalds@rtu.lv (R.R.); imants.adijans@rtu.lv (I.A.); 2 BMF Institute of Mechanical and Biomedical Engineering, Riga Technical University, 6A Kipsalas Street, LV-1048 Riga, Latvia; gatis.muiznieks_1@rtu.lv (G.M.); armands.leitans@rtu.lv (A.L.); 3 Faculty of Nuclear Sciences and Physical Engineering, Czech Technical University in Prague, Trojanova 13, 120 00 Prague, Czech Republic; jiri.capek@fjfi.cvut.cz (J.Č.); karel.trojan@fjfi.cvut.cz (K.T.); 4 Center of Competence “Smart Mechatronic, Eco- and Energy-Saving Systems and Technologies”, Technical University of Gabrovo, ul. “Hadji Dimitar” 4, 5300 Gabrovo, Bulgaria; pprodanov@tugab.bg; 5 Department of Machine Elements and Chemistry, Todor Kableshkov University of Transport, ul. Geo Milev 158, 1574 Sofia, Bulgaria; normunds.teirumnieks@vtu.bg

**Keywords:** laser surface hardening (LSH), high-power diode laser (HPDL), steel 42CrMo4, steel C45, prior heat treatment, case depth, microstructure, sustainable manufacturing

## Abstract

This study systematically investigates the laser surface hardening (LSH) behavior of two medium carbon steels—the low alloy 42CrMo4 and the plain carbon C45—using a 4 kW high power diode laser (HPDL). The influence of laser parameters (power: 3.0–3.8 kW; scanning speed: 10–16 mm/s), post-laser quenching medium (oil vs. air), and, critically, the initial material condition (normalized “raw” vs. quenched and tempered “Q&T”) on the case hardening depth (CHD) was evaluated. Hardness profiles defined the CHD at a threshold of 392 HV1, and microstructural analysis was conducted via optical microscopy. The results demonstrate that prior conventional Q&T heat treatment of 42CrMo4 enhances the subsequent laser-hardened depth by approximately 27% compared to laser treatment of the normalized material under identical parameters, providing a quantitative basis for process optimization. For Q&T 42CrMo4, the quenching medium had an insignificant effect on CHD, with air cooling proving equally effective as oil across the tested parameter range, offering an empirically validated route for sustainable processing. In contrast, C45 exhibited a substantially lower and less parameter-sensitive CHD, constrained by its inherent low hardenability. This comparative analysis underscores that hardening depth in 42CrMo4 is linearly controllable via energy input, whereas for C45 it is hardenability-limited. This work establishes that an integrated approach combining conventional bulk heat treatment with diode laser hardening using air cooling offers a highly effective, controllable, and sustainable surface engineering route for high-performance alloy steels.

## 1. Introduction

Surface engineering is a cornerstone of modern manufacturing, aimed at improving the surface properties of materials without compromising the properties of the base material [1]. Among various surface treatment techniques, laser surface hardening has emerged as a precise, flexible, and efficient thermomechanical process [2,3]. This technique utilizes a focused, high-energy laser beam to rapidly heat a thin surface layer of a ferrous alloy above its austenitization temperature (Ac3). Subsequently, the heated volume undergoes rapid self-quenching due to intense heat conduction into the cold bulk substrate. This fast cooling cycle, often exceeding the critical quenching rate for steel, facilitates a diffusionless transformation resulting in a hard, wear-resistant martensitic microstructure without the need for external liquid quenchants [4,5].

Compared to conventional through-hardening or induction hardening, laser hardening offers significant advantages. These include minimal part distortion and residual stress due to localized heat input, precise spatial control over the hardened zone geometry, negligible surface roughness changes, and high processing speeds [6,7]. This combination makes it particularly suitable for critical automotive components (e.g., camshafts, gear flanks, valve seats), tooling (dies, molds), and heavy machinery parts where dimensional stability and selective wear resistance are paramount [8]. Recent advances in laser surface hardening techniques and modeling approaches continue to expand these industrial applications [9].

The fundamental outcome of the laser hardening process—primarily the depth and hardness of the hardened zone—is governed by the thermal cycle imposed on the material. This cycle is critically dependent on the laser parameters, material properties (especially its hardenability and initial microstructure), and the characteristics of the laser source itself [10]. Therefore, establishing robust quantitative relationships between these factors and the resultant metallurgical characteristics is essential for the industrial adoption and optimization of this technology.

The materials selected for this study are the low-alloy steel 42CrMo4 (AISI 4140 equivalent) and the plain carbon steel C45 (AISI 1045 equivalent), representing two extremes in hardenability common in engineering. 42CrMo4, with additions of chromium (~1%) and molybdenum (~0.2%), exhibits significantly enhanced hardenability, allowing for deep martensite formation even at moderate cooling rates [11]. It is a preferred material for high-stress components such as shafts, gears, and connecting rods [12]. In contrast, C45, with approximately 0.45% carbon but no strong alloying elements, has limited hardenability, resulting in a steep hardness gradient and a hardened depth highly sensitive to cooling rate [13]. Nonetheless, its cost-effectiveness and good machinability make it ubiquitous for general engineering components [14]. A key, yet underexplored, factor is the role of the material’s initial condition prior to laser treatment. Recent studies suggest that a prior quenched and tempered (Q&T) microstructure, compared to a normalized state, can significantly enhance the efficiency and depth of subsequent laser hardening due to more favorable austenitization kinetics [15]. A comparative investigation of laser hardening on these two steels, considering their initial state, allows for a clear elucidation of the interplay between alloy composition, initial microstructure, and the process outcome.

The choice of laser source is a critical technological determinant. While CO_2_ and Nd:YAG lasers have been historically used, High-Power Diode Lasers (HPDLs) operating in the near-infrared range (λ ≈ 970–980 nm) have become dominant for surface hardening, especially in the multi-kilowatt range [16,17]. This shift is driven by principal advantages intrinsic to diode laser technology: superior absorption on bare metal surfaces (35–45% vs. 5–15% for CO_2_), eliminating the need for absorptive coatings [18]; an inherent homogenized “top-hat” beam profile that generates uniform temperature fields and minimizes soft zones [19,20]; and exceptional electrical efficiency (40–50%) leading to lower operational costs [21]. Recent comprehensive studies on HPDL surface transformation hardening of ferrous alloys have quantitatively confirmed these advantages for steels like 42CrMo4 and C45, highlighting HPDL’s superiority in achieving consistent and controllable hardening layers [22]. Furthermore, the high absorption and uniform beam often permit the use of simple compressed air cooling instead of liquid quenchants for sufficiently hardenable steels, aligning with goals of sustainable manufacturing [23]. In diode laser hardening with a static line beam, the primary controllable parameters are laser power P and scanning speed v, defining the linear energy density El = P/v, a key metric influencing heat input [24]. These parameters control the peak temperature, thermal field, and cooling rate [25]. The resulting hardened depth and hardness are direct consequences of this thermal cycle, determined by the volume of material transformed to martensite and its morphology [26]. While the general hierarchy of influencing factors (hardenability > initial microstructure > cooling intensity) is acknowledged, there is a distinct need for studies that provide precise quantitative data on the magnitude of these effects for specific HPDL-material combinations. Many existing models are based on Gaussian beams [27,28], and comprehensive comparative studies linking process parameters to final outcomes for both high- and low-hardenability steels, while also accounting for the critical variable of initial microstructure, are not exhaustive. Specifically, the quantitative benefit of a prior Q&T structure and the practical equivalence of air versus oil cooling for high-hardenability steels processed with modern HPDLs require further empirical validation to guide sustainable manufacturing [15,23]. Therefore, the primary objective of this study is to conduct a comprehensive experimental investigation into the diode laser hardening of 42CrMo4 and C45 steels. Using a 4 kW HPDL, this research aims to

(1) Quantify the relationships between laser parameters *P, v* and the resulting case hardening depth (CHD);
(2) Characterize the associated microstructural gradients;
(3) Evaluate and quantify the decisive influence of the initial material condition (normalized vs. Q&T) and empirically confirm the effect of the post-heat cooling medium (oil vs. air);
(4) Compare the process responses between the low-alloy and plain carbon steel, highlighting the role of composition and pretreatment in achieving controllable versus saturation-limited hardening.

The findings are intended to provide a robust empirical database, precise quantitative guidelines, and a validated sustainable processing route for optimizing diode laser hardening, facilitating its reliable implementation in industry.

## 2. Materials and Methods

### 2.1. Materials and Sample Preparation

This study employed two widely used medium-carbon steels to investigate the interplay between material composition, initial microstructure, and laser hardening response. The selected grades were the low-alloy 42CrMo4 (1.7225/AISI 4140) and the plain carbon C45 (1.0503/AISI 1045). 42CrMo4, with nominal additions of 1.0% Cr and 0.2% Mo, is known for its high hardenability and strength, making it suitable for deep-case hardening [11]. In contrast, C45 lacks significant alloying elements, resulting in limited hardenability, though it remains a cost-effective choice for surface wear resistance [14].

Chemical composition for 42CrMo4 and C45 steels was indicated by supplier in received material certificates. Chemical composition of steels was analyzed with glow discharge optical emission analyzer—Spectruma GDA550 (Spectruma Analytik GmbH, Hof, Germany)—and results were compared against received certificates (Table 1 and Table 2). No major discrepancies were detected between the certificates and the spectrographic analysis, and in both cases, the chemical compositions complied with the relevant standards (EN 10083-2 [29]; EN 10083-3 [30]).

Both materials were checked for the absence of significant surface decarburization before processing by preliminary microstructural examination near the edges of the samples. Test specimens for experiments for both steels 42CrMo4 and C45 were prepared from Ø30 mm bars with thickness of 20 mm. To achieve uniform surface roughness, specimens before laser hardening were ground using a belt polishing machine with abrasive papers of grit sizes P120, P240, P400, P600 and P800.

To isolate the effect of initial condition, samples were prepared in two distinct states: (i) a normalized (“raw”) condition for both steels, exhibiting a ferrite–pearlite microstructure, and (ii) a quenched and tempered (Q&T) condition for 42CrMo4 steel, consisting of a uniform tempered martensite structure obtained via conventional oil quenching and tempering.

Test specimens of C45 steel in normalized (“raw”) condition had surface hardness of 202–207 HV30 prior to laser hardening. Reached surface hardness of test specimens for 42CrMo4 steel after quenching and tempering was in the range of 423–430 HV30, while in as-received condition surface hardness was in the range of 303–308 HV30.

### 2.2. Diode Laser Hardening Procedure

Laser surface hardening was performed using a 4 kW high-power diode laser (HPDL) system operating at a wavelength of ~980 nm. The laser was configured to produce a continuous-wave beam with a rectangular “top-hat” intensity profile measuring 8 × 15 mm, ensuring uniform energy distribution across the treatment zone [22]. No absorptive coatings were applied, leveraging the high inherent absorptivity of ferrous metals at this wavelength.

The working distance was kept constant at 150 mm, and the laser head was fixed at a 0° incidence angle (normal to the sample surface). The beam profile was verified using a beam profiler prior to experimentation to confirm the ‘top-hat’ intensity distribution. A protective argon gas shield with a flow rate of 15 L/min was employed coaxially with the laser beam to prevent oxidation during processing.

The key processing variables were laser power P and scanning speed v. Power levels of 3.0, 3.5, and 3.8 kW were combined with scanning speeds ranging from 10 to 16 mm·s^−1^. The linear energy density *E_l_ = P/v*, a critical parameter governing heat input, was systematically varied to establish its correlation with the resulting hardened zone geometry [24].

### 2.3. Post-Laser Quenching

Immediately following laser irradiation, samples were subjected to controlled cooling. Two quenching media were employed: “oil quenching” using an industrial polymer quenchant (Serviscol 98SK-F1, BURGDORF GmbH, Stuttgart, Germany), 10% water), and “air quenching” via free convection in still air. This design allowed for a direct evaluation of the influence of cooling intensity on the final microstructure and case depth, particularly for the high-hardenability 42CrMo4 steel [23]. The oil quenching was performed by immersing the sample immediately (<2 s delay) after laser irradiation into a quench bath maintained at 40 °C. Air quenching was performed under laboratory ambient conditions (20–22 °C) with no forced convection. The temperature evolution during cooling for selected samples was monitored using a K-type thermocouple inserted 1 mm below the surface at the track centerline, confirming the cooling rate differential between the two media.

### 2.4. Metallographic and Mechanical Characterization

For analysis, hardened samples were sectioned transverse to the laser track, mounted, and prepared using standard metallographic techniques—samples were ground using silicon carbide abrasive papers with grit sizes of P320, P600 and P1200 on a Metkon Forcimat Forcipol 2V grinder–polisher, Bursa, Turkey, culminating in a final polish with 6 µm diamond suspension. The resulting microstructures were revealed by etching with 3% nital and examined using a Nikon Eclipse MA100N optical microscope (Tokyo, Japan) [22]. Microhardness profiling was conducted using a Vickers indenter (Qness A+) under a 1 kgf (HV1) load and a hardness measurement deviation of 3%. Hardness measurements were taken from the hardened surface down to the unaffected core material. The “case hardening depth (CHD)” was quantitatively defined as the distance from the surface to the point where the hardness value dropped to 392 HV1, providing a consistent and industrially relevant metric for comparison. The microhardness traverse was performed with a step size of 0.1 mm in the hardened zone and 0.2 mm in the transition region, ensuring accurate determination of the hardness gradient. The CHD was determined from the average of three separate profiles per sample, measured at the track centerline, to ensure statistical robustness. Statistical analysis (*t*-test, α = 0.05) confirmed no significant difference in CHD between air and oil quenching across all parameter sets for Q&T samples.

Optional addition on phase analysis: X-ray diffraction (XRD) analysis was performed on the surface of selected samples using Cu-Kα radiation to qualitatively assess the presence of retained austenite in the hardened layer [28].

## 3. Results and Discussion

### 3.1. Parametric Control and Material-Dependent Response

The experimental results clearly delineate the contrasting behaviors of 42CrMo4 and C45 steels under diode laser hardening. The influence of linear energy density El = P/v was found to be the dominant thermal parameter governing the hardened depth for the high-hardenability steel, while the response of C45 was largely saturated due to its metallurgical constraints.

**Figure 1:**
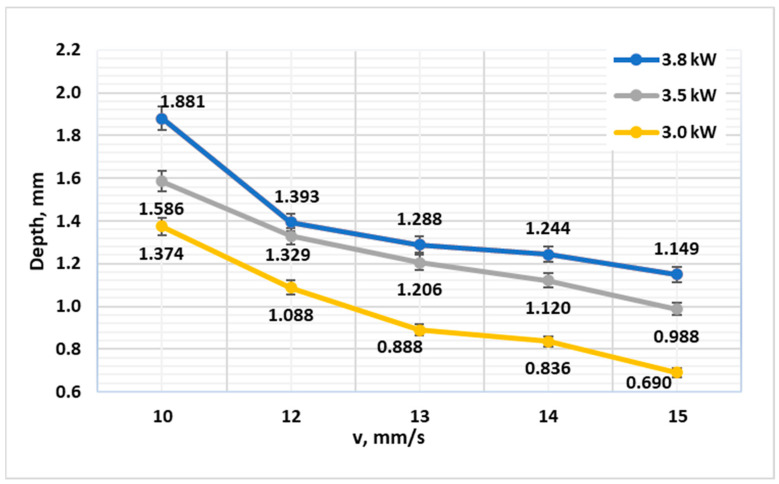
Diagram of the reached depth of laser hardening depending on the laser processing speed at powers of 3.0 kW, 3.5 kW and 3.8 kW for steel 42CrMo4 (Q&T, oil quenched).

For 42CrMo4 in the Q&T condition, a strong and predictable relationship between processing parameters and hardened depth was observed (Figure 1). As anticipated, increasing the linear energy density—either by raising the laser power or reducing the scanning speed—consistently produced a deeper hardened case. For instance, at a fixed speed of 10 mm·s^−1^, the depth increased progressively from 1.38 mm at 3.0 kW to 1.89 mm at 3.8 kW. A near-linear correlation between El and CHD was observed for 42CrMo4 in the Q&T condition, with a regression slope of approximately 0.18 mm per kJ/m over the tested range. This consistent trend highlights the controllability of the HPDL process for tailored case depths in alloy steels, confirming recent findings on the effectiveness of HPDL for ferrous alloys [22].

**Figure 2:**
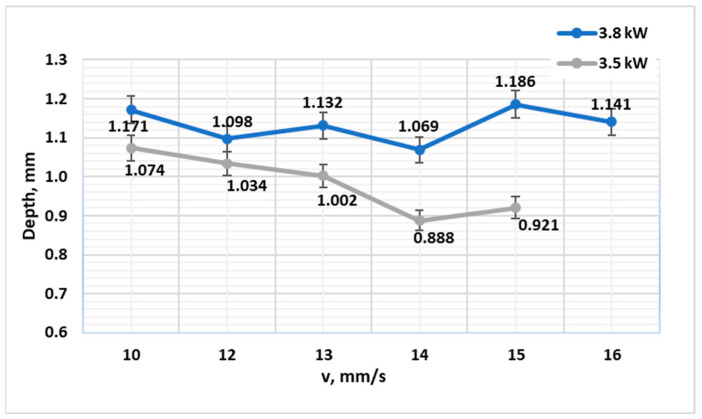
Diagram of the reached depth of laser hardening depending on the laser processing speed at powers of 3.0 kW, 3.5 kW, and 3.8 kW for steel C45 (Q&T, air quenched).

In stark contrast, C45 steel exhibited a markedly different response (Figure 2). The achievable hardening depth was confined to a narrow range of approximately 0.9–1.2 mm across the entire matrix of tested powers and speeds. This plateau effect indicates that the process outcome is governed not by the input energy, but by the material’s intrinsic “hardenability limit” [13]. The absence of alloying elements like Cr and Mo in C45 necessitates extremely high cooling rates to form martensite, which are only attained in a shallow subsurface layer during self-quenching, thereby constraining the maximum case depth. This hardenability-limited regime was further confirmed by the minimal variation in CHD (<0.3 mm) across the entire *E_l_* range of 187.5–380 J/mm. Even at the highest energy input (3.8 kW, 10 mm/s), the cooling rate beyond ~1.2 mm depth fell below the critical rate for martensite formation in C45, resulting in a mixed microstructure of martensite, bainite, and pearlite in the transition zone.

**Figure 3:**
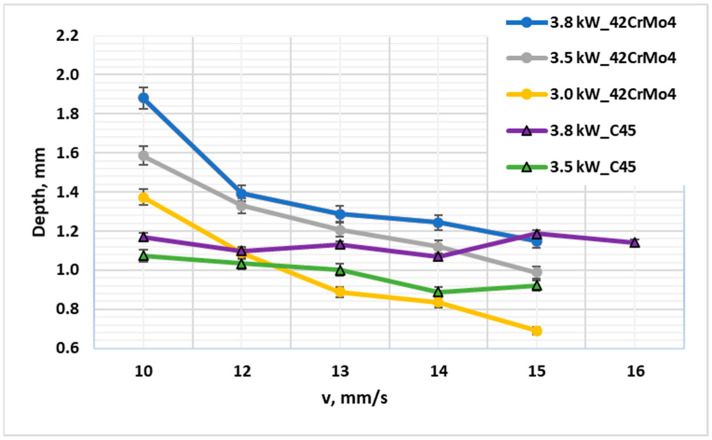
Diagram showing the achieved laser hardening depth as a function of laser processing speed at laser powers of 3.0 kW, 3.5 kW, and 3.8 kW for 42CrMo4 steel (oil quenched) and C45 steel (air quenched).

**Figure 4:**
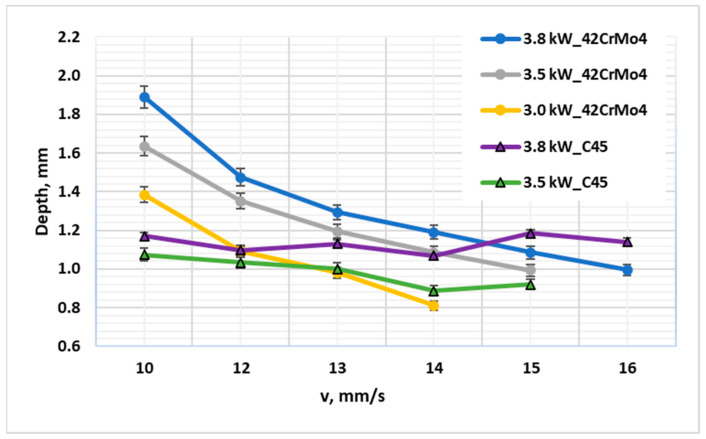
Diagram showing the achieved laser hardening depth as a function of laser processing speed at laser powers of 3.0 kW, 3.5 kW, and 3.8 kW for 42CrMo4 and C45 steels, both air quenched.

A direct comparative analysis reinforces this conclusion (Figure 3 and Figure 4). Under identical laser parameters (e.g., 3.8 kW, 10 mm·s^−1^), 42CrMo4 developed a case depth (~1.90 mm) nearly 60% greater than that of C45 (~1.18 mm). For example, at El = 380 J/mm (3.8 kW, 10 mm/s), the CHD for 42CrMo4 was 1.90 mm compared to 1.18 mm for C45—a 61% increase. This substantial difference persisted even at lower energy densities, with the gap narrowing only slightly as El decreased, underscoring the dominant role of alloy composition [11]. Surface hardness values also reflected this divergence: 42CrMo4 consistently achieved surface hardness >700 HV1, while C45 reached a maximum of ~650 HV1, with greater scatter due to its sensitivity to local cooling variations. Crucially, this performance gap persisted even when both materials were air-cooled, demonstrating that the superiority of 42CrMo4 is rooted in its fundamental metallurgy, not merely in the potential for more aggressive post-heat quenching.

Thus, while diode laser hardening depth in 42CrMo4 can be precisely tuned via El across a wide range, the process for C45 is essentially depth-saturated, making it suitable only for applications where shallow case depths (≤1.2 mm) are acceptable.

### 3.2. The Critical Role of Initial Microstructure and Quenching Medium

A pivotal finding of this work is the significant impact of the steel’s initial condition prior to laser treatment. For 42CrMo4, samples with a prior Q&T microstructure demonstrated a substantial advantage. When processed at 3.8 kW and 10 mm·s^−1^, the Q&T material achieved a CHD of approximately 1.88–1.89 mm, whereas the normalized (“raw”) material reached only about 1.37–1.38 mm—an enhancement of roughly 27%. This improvement corresponds to an increase in effective austenitization depth of ~0.5 mm under the same thermal cycle, directly attributable to the more favorable starting microstructure.

This improvement is due to the kinetics of austenitization. The fine, dispersed carbides within the relaxed martensite of the Q&T structure dissolve more rapidly and completely during the short laser treatment process compared to the coarser carbides in the normalized ferrite-pearlite structure [15]. The fine, dispersed carbides within the relaxed martensite of the Q&T structure dissolve more rapidly and completely during the short laser treatment process compared to the coarser carbides in the normalized ferrite-pearlite structure [14,28]. The finer initial carbide structure provides a larger interfacial area for carbon diffusion, accelerating the formation and retention of austenite. This more efficient phase transformation allows the austenitization front to penetrate deeper into the material for the same energy input, resulting in a thicker layer capable of transforming to martensite upon cooling. Microstructural analysis confirms that the prior Q&T condition also reduces the presence of untransformed ferrite in the hardened zone, contributing to a more uniform high-hardness plateau near the surface.

**Figure 5:**
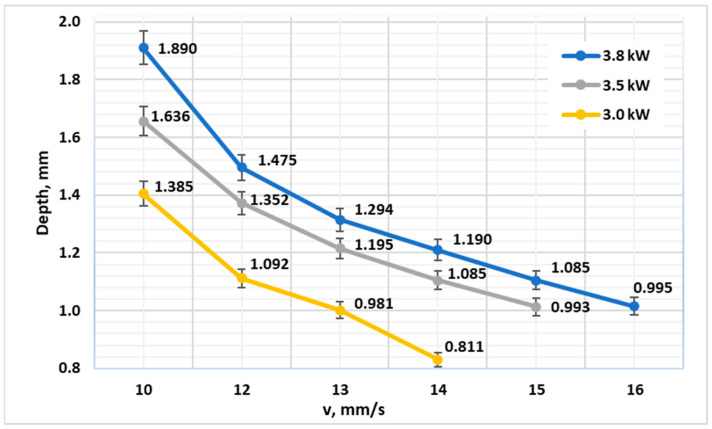
Diagram showing the achieved laser hardening depth as a function of laser processing speed at laser powers of 3.0 kW, 3.5 kW, and 3.8 kW for 42CrMo4 steel in the quenched and tempered (Q&T), air quenched condition.

**Figure 6:**
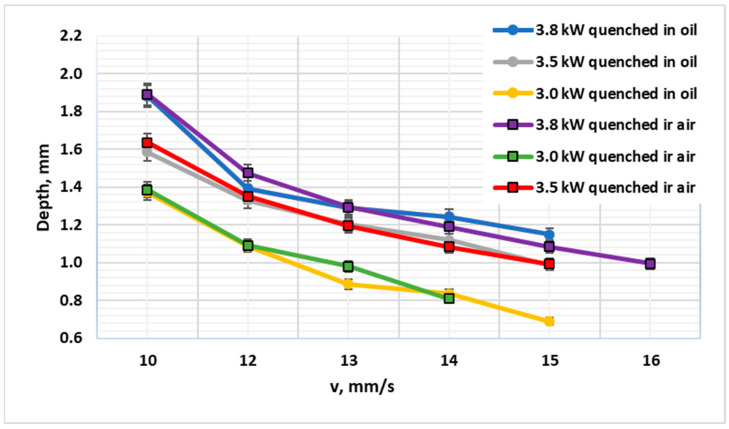
Diagram showing the achieved laser hardening depth as a function of laser processing speed at laser powers of 3.0 kW, 3.5 kW, and 3.8 kW, comparing oil and air quenching for quenched and tempered (Q&T) 42CrMo4 steel.

Equally significant is the finding regarding the post-laser quenching medium for 42CrMo4. For specimens in the Q&T condition, the choice between oil and air quenching had a negligible effect on the final CHD (Figure 5 and Figure 6). Both methods yielded statistically identical depths at high energy inputs. Statistical analysis (*t*-test, α = 0.05) confirmed no significant difference in CHD between air and oil quenching across all parameter sets for Q&T 42CrMo4. This result can be directly linked to the steel’s high hardenability imparted by Cr and Mo. These alloying elements shift the continuous cooling transformation (CCT) diagram to longer times, dramatically lowering the critical cooling rate required for martensite formation [11]. For 42CrMo4, the critical cooling rate to avoid pearlite/bainite formation is below 30 °C/s, which is readily achieved even with still-air cooling (~50–100 °C/s in the near-surface region) following laser heating. Consequently, even the relatively moderate cooling rate provided by still air is sufficient to induce a full martensitic transformation throughout the austenitized depth. This finding is consistent with recent studies on laser hardening under air and water environments, which confirm that for sufficiently hardenable steels, air cooling provides adequate transformation rates [23].

This insight carries considerable practical importance, as it validates the use of a simpler, cleaner, and more sustainable air-cooling process without compromising the technical outcome for properly pre-treated alloy steels. From a sustainability perspective, replacing oil quenching with air cooling eliminates quenchant disposal, reduces Volatile Organic Compounds (VOC) emissions, and lowers process complexity and cost [15].

**Figure 7:**
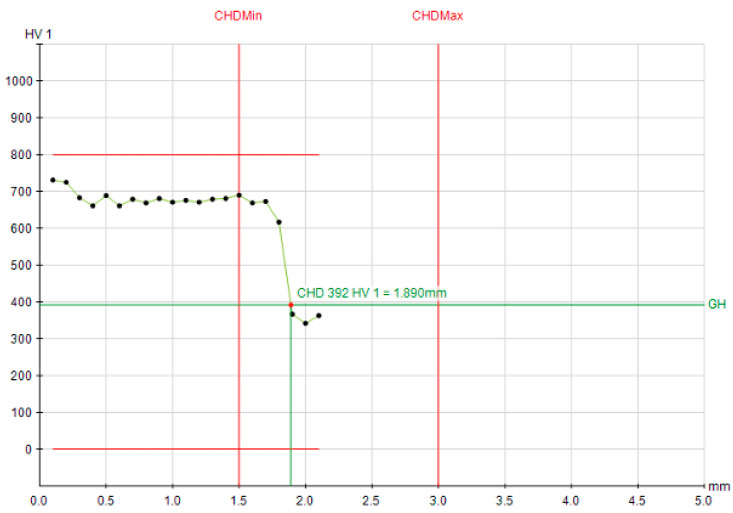
Hardness depth profile for air quenched Q&T 42CrMo4 steel; CHD at 392 HV1 = 1.890 mm.

**Figure 8:**
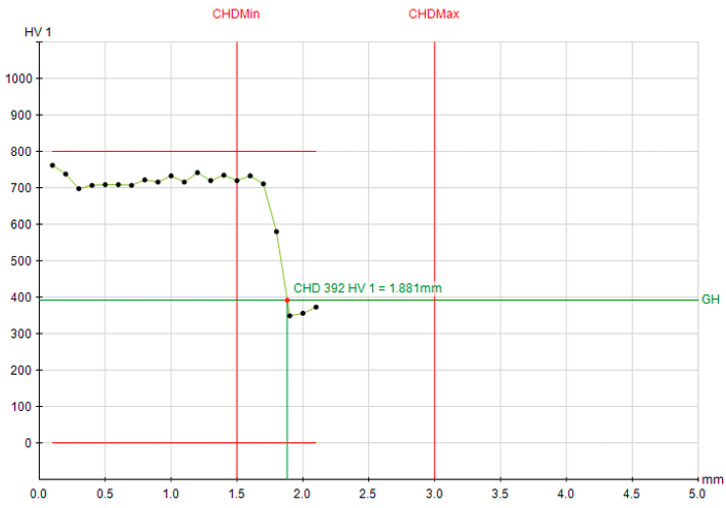
Hardness depth profile for oil quenched Q&T 42CrMo4 steel; CHD at 392 HV1 = 1.881 mm.

**Figure 9:**
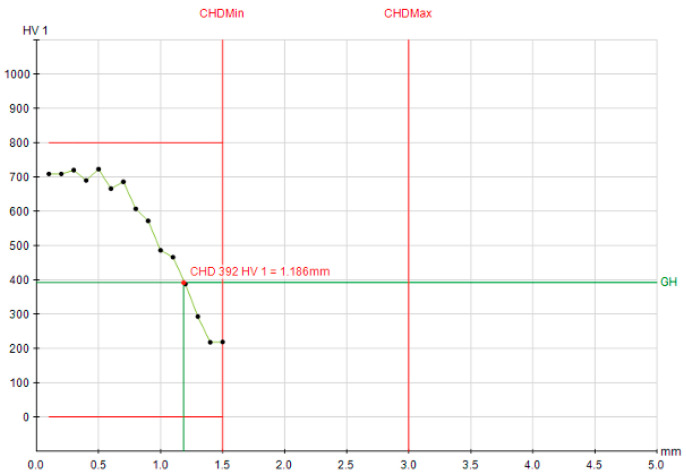
Hardness depth profile for air quenched C45 steel; CHD at 392 HV1 = 1.186 mm.

The corresponding microhardness profiles provide a visual confirmation of these trends (Figure 7, Figure 8 and Figure 9). The profiles for Q&T 42CrMo4, whether air- or oil-quenched, are nearly superimposable, showing a high surface hardness (>700 HV1) and a gradual decline to the 392 HV1 threshold at a depth of ~1.88 mm. The hardness gradient (dHV/dx) in the transition zone was measured at ~180 HV/mm for 42CrMo4, reflecting its good hardenability and deep case capability. The profile for C45, however, is much steeper, reaching the same hardness threshold at only 1.19 mm, graphically illustrating its limited hardenability. For C45, the hardness gradient exceeded 350 HV/mm, indicating a sharp transition and limited depth of effective hardening. This stark contrast visually reinforces the hierarchy of influencing factors: material hardenability > initial microstructure > cooling intensity.

### 3.3. Microstructural Evidence

**Figure 10:**
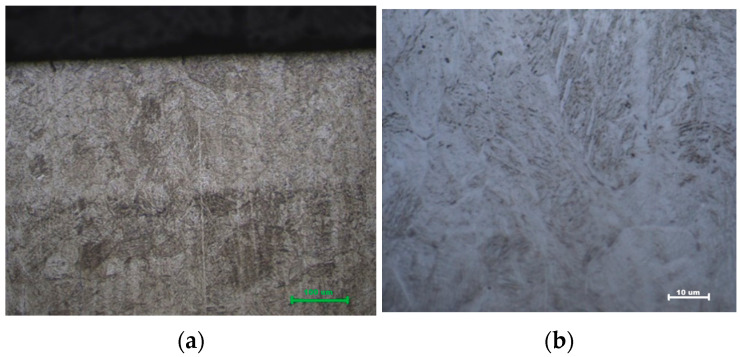
Martensitic microstructure of quenched and tempered (Q&T) 42CrMo4 steel after laser hardening and air quenching: (**a**) 500× and (**b**) 1000× magnification.

**Figure 11:**
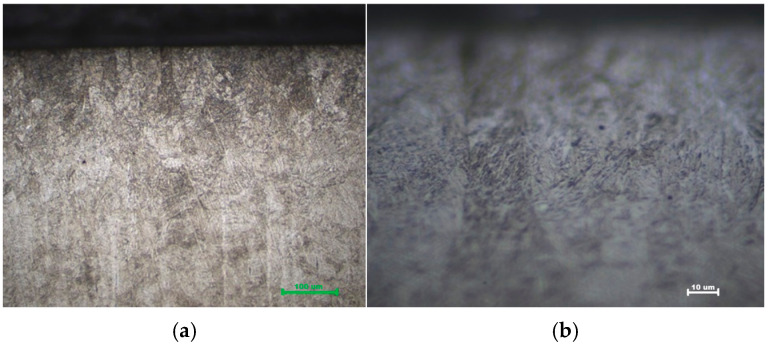
Martensitic microstructure of quenched and tempered (Q&T) 42CrMo4 steel after laser hardening and oil quenching (**a**) 500× and (**b**) 1000× magnification.

Microstructural examination substantiates the mechanical data. Optical micrographs of the hardened zone in Q&T 42CrMo4 reveal a characteristic fine, acicular martensitic structure for both air- and oil-cooled samples (Figure 10 and Figure 11). The martensite lath size ranged between 1 and 5 μm, with no significant difference observed between air and oil quenching, confirming that cooling rate did not markedly affect the final martensite morphology in this alloy. The uniformity and depth of this transformed zone correspond directly to the measured hardness profiles and case depths. At the interface between the hardened zone and the substrate, a narrow (~50–100 μm) transition region containing tempered martensite from the prior Q&T structure was observed, confirming the precise localization of the thermal cycle [22].

**Figure 12:**
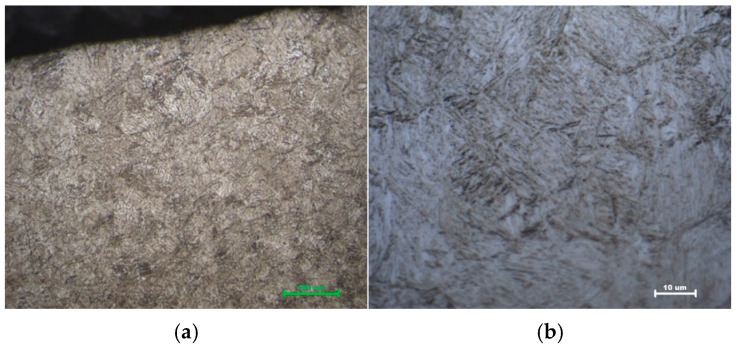
Martensitic microstructure of quenched and tempered (Q&T) C45 steel after laser hardening and air quenching (**a**) 500× and (**b**) 1000× magnification. The micrograph clearly shows the shallow martensitic case (lighter etching region) above the darker, untransformed ferrite–pearlite core.

For C45, the martensitic layer is visibly thinner and less uniform (Figure 12), consistent with its rapid hardness drop-off and the constraints imposed by its lower hardenability and carbon content. In addition to the shallower martensitic case, the microstructure of C45 frequently exhibited evidence of partial transformation, including isolated patches of ferrite and pearlite within the nominally hardened zone, especially at greater depths and in lower-energy processing conditions. This microstructural inhomogeneity directly explains the greater scatter in surface hardness values and the steeper hardness gradient compared to 42CrMo4.

Furthermore, for both steels, the prior microstructure influenced martensite morphology. In Q&T 42CrMo4, the fine prior tempered martensite resulted in a similarly refined laser-hardened martensite. In contrast, the normalized C45, with its coarser initial ferrite–pearlite structure, produced a more irregular martensitic matrix with less consistent lath orientation. These observations reinforce the conclusion that initial microstructure not only affects the depth of hardening but also the quality and homogeneity of the hardened layer [15] (Table 3).

### 3.4. Synthesis and Technological Implications

The collective results of this study underscore a consistent hierarchy of influencing factors for diode laser hardening: material composition (hardenability) > initial microstructure > cooling intensity. Beyond confirming this hierarchy, our work provides specific quantitative insights and practical validations that advance the state of the art for HPDL processing:

Material Selection and Parametric Sensitivity are Foundational: For applications demanding deep, reliable case depths with high parametric controllability, alloy steels such as 42CrMo4 are essential. Its inherent hardenability, provided by chromium and molybdenum, ensures that significant hardened depths (up to ~1.9 mm) can be achieved and finely tuned through laser parameters [22]. In contrast, plain carbon steels like C45 are inherently limited by their low hardenability, resulting in a maximum effective case depth of ~1.2 mm with high sensitivity to cooling conditions [13]. Therefore, C45 is suitable only for applications where shallow case hardening is acceptable and process conditions are tightly controlled.

Prior Heat Treatment is a Powerful Lever: Implementing a conventional quench and temper (Q&T) cycle before laser hardening is not merely a preparatory step but a “performance multiplier.” For 42CrMo4, starting from a Q&T microstructure increased the laser-hardened depth by approximately 27% (from ~1.38 mm to ~1.89 mm) compared to the normalized condition under identical high-energy parameters (3.8 kW, 10 mm/s). This significant enhancement provides a clear quantitative argument for integrating bulk heat treatment into the manufacturing chain for critical components. This result is consistent with and quantitatively extends the results of studies by other authors, providing a specific magnitude for the achievable efficiency increase with HPDL on pre-treated alloy steel [15]. 

Cooling Simplification is Empirically Validated for Sustainable Manufacturing: The experimental equivalence of air and oil quenching for Q&T 42CrMo4, confirmed by statistical analysis (*t*-test, α = 0.05) across all parameter sets, carries significant practical implications. The high hardenability of this steel lowers the critical cooling rate required for martensite formation, making still-air cooling sufficient to achieve full transformation throughout the austenitized depth. This finding aligns with recent investigations into laser hardening under air and water environments, which demonstrate the viability of simplified cooling approaches for sufficiently hardenable materials [23]. This enables the design of more sustainable, cost-effective, and cleaner manufacturing cells by eliminating the need for quench oils, associated filtration systems, waste disposal, and post-process cleaning operations [15].

These insights converge to propose an optimized and sustainable processing route for high-performance components: “Conventional Bulk Q&T → Diode Laser Surface Hardening → Air Cooling”. This integrated approach delivers multiple synergistic benefits:

- Maximized Component Life: A deep, wear-resistant martensitic case is combined with a tough, tempered martensite core;
- Process Efficiency and Precision: The energy efficiency, beam uniformity, and precise control of High-Power Diode Lasers (HPDLs) are fully leveraged [22];
- Reduced Environmental Impact: Eliminating quench oils lowers the carbon footprint, minimizes hazardous waste, and aligns with green manufacturing principles [15];
- Operational Simplicity and Cost Reduction: Air cooling simplifies plant layout, reduces maintenance, and lowers operational costs compared to liquid quenching systems [23].

This strategy represents a tangible advancement toward sustainable, circular manufacturing practices within the automotive, tooling, and heavy machinery sectors. It provides a concrete, quantitatively supported model for implementing environmentally conscious surface engineering without sacrificing performance, as outlined in recent comprehensive reviews of laser surface hardening techniques [9]

## 4. Conclusions

This systematic experimental study on the diode laser hardening of 42CrMo4 and C45 steels leads to the following conclusive points:

1. Prior conventional quench and temper (Q&T) heat treatment of 42CrMo4 steel provides a quantifiable and significant benefit, increasing the depth of the subsequent laser hardened zone by approximately 27% compared to laser processing of normalized material under identical parameters. This enhancement is attributed to the more rapid and efficient austenitization kinetics of the fine, carbide-strengthened tempered martensite starting microstructure [15].
2. For 42CrMo4 in the Q&T condition, the post-laser quenching medium (oil vs. air) has a negligible impact on the achieved case hardening depth, as statistically validated across the tested parameter range. The high hardenability imparted by chromium and molybdenum lowers the critical cooling rate, making still air cooling sufficient for a complete martensitic transformation, thereby enabling a more sustainable process [23].
3. Material hardenability is the primary determinant of achievable CHD and parametric sensitivity. The low-alloy 42CrMo4 achieves significantly greater (up to ~60% deeper), more controllable, and linearly energy-dependent case depths [22]. In contrast, the hardening depth in plain carbon C45 is limited to ~1.2 mm due to its intrinsic hardenability ceiling, exhibiting a saturation effect regardless of energy input [13].
4. An optimal, sustainable processing route is identified and validated: For components demanding superior wear resistance and longevity, the integrated sequence of “conventional bulk Q&T → diode laser surface hardening → air cooling” for 42CrMo4 offers a highly effective, resource-efficient, and environmentally friendly manufacturing strategy. This route, combining deep and controllable case hardening with oil-free cooling, represents a key practical outcome of this research, aligning with the goals of sustainable and circular industrial production as highlighted in recent advances in the field [9].

These findings provide essential quantitative knowledge and validated guidelines for selecting materials, designing heat treatment sequences, and optimizing laser parameters to maximize the benefits of diode laser hardening technology for enhanced component durability and manufacturing sustainability.

## Figures and Tables

**Table 1:** Chemical composition of 42CrMo4 steel (wt.%).

Element	C	Si	Mn	P	S	Cr	Mo
Content	0.43	0.25	0.75	0.02	0.02	1.05	0.22

**Table 2:** Chemical composition of C45 steel (wt.%).

Element	C	Si	Mn	P	S
Content	0.46	0.25	0.65	0.03	0.03

**Table 3:** Comparison on key results from this survey with selected literary data for laser hardening on medium carbon steels.

Source (Reference)	Material (Condition)	Laser Source/Power	Max. CHD [mm]	Max. Surface Hardness	Key Observed Effect/Focus
Present study	42CrMo4 (Q&T)	HPDL, 4 kW, Top-hat	~1.89	>700 HV1	27% increase of CHD from Q&T pre-processing; equivalence air/oil cooling.
Present study	42CrMo4 (Normal)	HPDL, 4 kW, Top-hat	~1.38	>650 HV1	Basic line for the effect on preliminary processing.
Present study	C45 (Normal)	HPDL, 4 kW, Top-hat	~1.18	~650 HV1	Limited CHD from malleability; weak parametric sensitivity.
Telasang and [11]	42CrMo4	Nd:YAG, 3.5 kW	1.2–1.8	~650–750 HV	Comparatively study on malleability and efficiency of the process.
Mousavi and [14]	AISI 4140 (42CrMo4 equiv)	Diode laser	Increased *	Increased *	Significant improvement at laser hardening from Q&T pre-processing.
Gehricke and [21]	42CrMo4	CO_2_ vs. HPDL	Comparable	Comparable	HPDL provides more even and controllable hardened layers compared to CO_2_.
Rodriguez and [22]	42CrMo4	HPDL	Comparable at air/oil	Comparable at air/oil	Rating on sustainability on air cooling as alternative on oil.

## Data Availability

The original contributions presented in this study are included in the article. Further inquiries can be directed to the corresponding author.

## Abbreviations

The following abbreviations are used in this manuscript:

LSH	Laser Surface Hardening
HPDL	High-Power Diode Laser
Q&T	Quenched and Tempered
CHD	Case Hardening Depth
CCT	Continuous Cooling Transformation
XRD	X-ray Diffraction
VOC	Volatile Organic Compounds
